# A Virtual Multidisciplinary Care Program for Management of Advanced Chronic Kidney Disease: Matched Cohort Study

**DOI:** 10.2196/17194

**Published:** 2020-02-12

**Authors:** Paulina Kaiser, Olivia Pipitone, Anthony Franklin, Dixie R Jackson, Elizabeth A Moore, Christopher R Dubuque, Carmen A Peralta, Anthony C De Mory

**Affiliations:** 1 Samaritan Health Services Corvallis, OR United States; 2 Cricket Health San Francisco, CA United States; 3 Kidney Health Research Collaborative at the University of California San Francisco San Francisco, CA United States

**Keywords:** chronic kidney disease, end-stage renal disease, online social networking, patient education, renal dialysis

## Abstract

**Background:**

It is not well established whether a virtual multidisciplinary care program for persons with advanced chronic kidney disease (CKD) can improve their knowledge about their disease, increase their interest in home dialysis therapies, and result in more planned outpatient (versus inpatient) dialysis starts.

**Objective:**

We aimed to evaluate the feasibility and preliminary associations of program participation with disease knowledge, home dialysis modality preference, and outpatient dialysis initiation among persons with advanced CKD in a community-based nephrology practice.

**Methods:**

In a matched prospective cohort, we enrolled adults aged 18 to 85 years with at least two estimated glomerular filtration rates (eGFRs) of less than 30 mL/min/1.73 m2 into the Cricket Health program and compared them with controls receiving care at the same clinic, matched on age, gender, eGFR, and presence of heart failure and diabetes. The intervention included online education materials, a virtual multidisciplinary team (nurse, pharmacist, social worker, dietician), and patient mentors. Prespecified follow-up time was nine months with extended follow-up to allow adequate time to determine the dialysis start setting. CKD knowledge and dialysis modality choice were evaluated in a pre-post survey among intervention participants.

**Results:**

Thirty-seven participants were matched to 61 controls by age (mean 67.2, SD 10.4 versus mean 68.8, SD 9.5), prevalence of diabetes (54%, 20/37 versus 57%, 35/61), congestive heart failure (22%, 8/37 versus 25%, 15/61), and baseline eGFR (mean 19, SD 6 versus mean 21, SD 5 mL/min/1.73 m2), respectively. At nine-month follow-up, five patients in each group started dialysis (*P*=.62). Among program participants, 80% (4/5) started dialysis as an outpatient compared with 20% (1/5) of controls (OR 6.28, 95% CI 0.69-57.22). In extended follow-up (median 15.7, range 11.7 to 18.1 months), 19 of 98 patients started dialysis; 80% (8/10) of the intervention group patients started dialysis in the outpatient setting versus 22% (2/9) of control patients (hazard ratio 6.89, 95% CI 1.46-32.66). Compared to before participation, patients who completed the program had higher disease knowledge levels (mean 52%, SD 29% versus mean 94%, SD 14% of questions correct on knowledge-based survey, *P*<.001) and were more likely to choose a home modality as their first dialysis choice (36%, 7/22 versus 68%, 15/22, *P*=.047) after program completion.

**Conclusions:**

The Cricket Health program can improve patient knowledge about CKD and increase interest in home dialysis modalities, and may increase the proportion of dialysis starts in the outpatient setting.

## Introduction

Care of persons with kidney disease represents an enormous health and economic burden in the United States, with expenditures over $114 billion in costs to Medicare alone [1]. Persons with advanced chronic kidney disease (CKD) have high rates of hospitalization and cardiovascular morbidity, mortality, and premature death [2]. Up to 35% of persons who begin dialysis have little or no nephrology care before reaching end-stage renal disease (ESRD), and up to half of those with ESRD “crash” into dialysis in an unplanned and costly acute care setting [1]. Despite the availability of home dialysis therapies, such as peritoneal dialysis and home hemodialysis, which are associated with higher quality of life and potentially lower costs [3,4], only 12% of patients begin dialysis at home [1]. The urgency of improving outcomes and reducing costs is highlighted by the Advancing Kidney Health executive order signed on July 10, 2019, which aims to reform the payment structure for kidney care.

Studies show that one of the most effective strategies to improve outcomes and reduce costs for persons with ESRD is to provide multidisciplinary care and education for high-risk persons at earlier stages of CKD [5]. One randomized trial showed that a multidisciplinary care program aimed at caring for persons with stages 4 and 5 CKD reduced hospitalizations and increased use of transplant and home dialysis modalities [6]. Another randomized trial in Canada and Europe showed that multidisciplinary care of patients with stages 3 and 4 CKD was associated with a 20% reduction in the incidence of a composite renal endpoint including death, ESRD, and 50% increase in serum creatinine [7]. Randomized trial evidence [8] and several observational studies [9] have shown that education programs that include multidisciplinary teams increase the proportion of patients choosing home dialysis modalities.

However, these multidisciplinary care programs require tremendous time commitment, cost, and personnel. The use of technology could allow for more scalable interventions at lower costs and with further reach. We previously showed that an online digital education program for advanced CKD was feasible to deploy and effective in increasing self-efficacy, knowledge, and the probability of choosing a home dialysis modality [10]. However, whether a virtual program can be extended to include multidisciplinary care is not well known. The association of virtual program use for management of persons with advanced CKD with clinical outcomes is less established. Research in this space is limited by several factors, including the need for large-scale studies in nonacademic settings that require substantial resources, detailed assessments to ensure intervention fidelity, and long follow-up periods [11]. It is also not well established whether electronic health records (EHRs) can be used to accurately and systematically track kidney disease-related outcomes, including incident dialysis, modality of dialysis starts, and outpatient dialysis starts [12-14].

We designed this study to assess the feasibility of deploying a virtual multidisciplinary care program for the management of advanced CKD in a community-based nephrology clinic and evaluate the association between program participation and patient disease knowledge, dialysis modality preference, and outpatient dialysis initiation rates.

## Methods

### Setting and Consent

This study has two components: a prospective matched cohort and a pre-post survey among participants in the intervention group.

Participants were recruited between November 2017 and May 2018 through Samaritan Kidney Specialists, a community-based adult nephrology clinic based in Corvallis, Oregon, with four nephrologists. All participants in the intervention group signed an informed consent form. Study approval and a waiver of documentation of informed consent from the matched comparison group members were obtained by the Samaritan institutional review board.

### Intervention Group

Patients were eligible for the intervention group if they had at least one routine encounter with the study nephrologist (AD) in the previous six months, had two estimated glomerular filtration rate (eGFR) results less than 30 mL/min/1.73 m^2^ measured at least three months apart, were aged 18 to 85 years, spoke English, had access to a computer or mobile phone with internet access, and reported being comfortable using email. Exclusion criteria included current dialysis treatment, a previous kidney transplant, hospice care, a life expectancy of less than nine months as determined by the nephrologist, and any other clinically significant condition that would interfere with engagement with the study or their ability to provide informed consent (ie, dementia). Patients with scheduled appointments with the study nephrologist were screened for eligibility using their EHRs. The nephrologist then introduced the study to eligible patients during the appointment; interested patients met with a research coordinator immediately afterward to enroll and subsequently received an email invitation to join the program. Program staff would attempt to contact patients by phone if they did not respond to the email within three days. Patients did not receive any guidance on how to use the program and were told to engage if and when they wanted to. Because this was designed as a pilot study and power was a secondary consideration, our intended sample size for the intervention group was 50 participants.

### Comparison Group

We intended to include two matched comparators for each intervention participant. Matched comparators were identified using their EHRs, and they had to have at least one routine encounter at the same nephrology clinic with any of the nephrologists in the past six months, have two eGFR tests less than 30 mL/min/1.73 m^2^ measured at least three months apart, and be aged 18 to 85 years. Comparators were matched based on age (± 10 years), gender, last eGFR value (± 10 points), diabetes status (yes or no), and congestive heart failure status (yes or no).

### The Cricket Health Virtual Chronic Kidney Disease Care Program

The two-part Cricket Health virtual CKD care online program includes education, modality decision modules, and access to a nurse, dietitian, pharmacist, social worker, and peer mentors for patient education, monitoring, and support of clinical goals established by the nephrologist. The first component is a multimodal educational program that incorporates videos related to CKD and its complications. Informed by prior work [10], it also includes details on modality choices for ESRD therapy (in-home peritoneal dialysis, in-home hemodialysis, in-center hemodialysis, transplant, or conservative care). We have previously described the educational component in detail [12]. In brief, the module includes written materials in the form of frequently asked questions, short videos, and chat features with a nurse, patient mentors, and peer patients. The duration of time in this phase varies based on a participant’s level of interaction and willingness to decide on a preferred treatment modality.

An additional component of the program is condition management. In this phase, the ancillary team supports the nephrologist-established clinical goals. The nephrologist first documents the clinical goals related to target blood pressure, weight, dietary counseling needs, medications, and dialysis access planning as appropriate, and the multidisciplinary team then supports these goals. The team also provides social support and continued education and reinforcement of key knowledge about kidney disease. For example, the nurse and pharmacist may work on education about hypertension and ensure medication reconciliation with the patient and then make recommendations to the physician. The pharmacist may also teach the patients about medications and the importance of adherence. The nutritionist may provide education and sample meals for low-sodium goals or reduced potassium intake. The ancillary team can also help patients transition to dialysis by educating them about permanent access procedures. These goals may be set at any point after study enrollment and are updated as needed. To support the patient in achieving all goals, the condition management phase includes additional educational videos and access to an online chat with a social worker, pharmacist, or dietician in addition to the nurse, patient mentors, and peer patients from the previous phase. Clinicians interact with patients through a proprietary Cricket Health online platform. The interaction with the nephrologist can be via fax or telephone.

### Survey of Intervention Patients

Intervention participants completed surveys about their knowledge of dialysis modalities, confidence in managing dialysis, and satisfaction with the online platform. Survey questions were adapted from prior studies [15-18] that we have previously published [10] (survey questions are available in Multimedia Appendix 1). The prestudy survey was completed in person after study enrollment; the posteducation survey was completed via email after the educational phase. The average time from completion of the prestudy to posteducation survey was 67 days (range 11-185 days).

### Baseline Clinical Data Elements

Demographic and baseline clinical information, including age, gender, race, ethnicity, insurance status, comorbidities, A_1c_, albumin, GFR, blood pressure, use of statin or inhibitors of the renin-angiotensin system (RAS), and number of nephrology visits, was obtained from Samaritan’s EHR system (Epic). Laboratory values (A_1c_, albumin, GFR, blood pressure) were included if they were recorded within 90 days before baseline (the value recorded closest to baseline was used). Statin and RAS inhibitor use were determined based on prescriptions placed within three months of baseline. Comorbidities (congestive heart failure, chronic obstructive pulmonary disease, and coronary artery disease) were identified based on encounters, billing, or active problem diagnoses within the EHR using *ICD-10* codes. The day that patients first logged in to the program was used as the baseline date for clinical data; patients in the comparison group were given the same baseline date as their matched intervention.

### Outcomes

The primary clinical outcome of this study was outpatient dialysis start at nine-month follow-up, defined as having a first treatment of chronic dialysis in the outpatient setting. We initially planned to collect dialysis start data from a systematic chart review of the EHR conducted by nonclinical staff to record relevant encounters, diagnosis codes, and procedure codes. However, we were unable to validate the accuracy of this approach. Therefore, we developed and incorporated a physician-adjudication process whereby a physician (CD), who was blinded to the intervention assignment and was not part of the practice, reviewed charts and identified dialysis starts during the study period and details of that start (modality, setting, planned or unplanned). In cases of uncertainty, the study nephrologist reviewed the case (AD). Secondary outcomes included mortality and kidney transplant status. Due to the delay with the physician-adjudication process, we were able to extend follow-up substantially. We present results at nine months (prespecified) and with the full follow-up (median 15.7, range 11.7-18.1 months) as a post hoc analysis.

### Analytic Methods

We used a pre-post design to compare survey results from before and after the program educational phase for the intervention participants using a Wilcoxon signed rank test for the average percent correct on seven knowledge-based questions and an exact symmetry test for intended type of dialysis. McNemar chi-square tests were used to assess changes in fear, confidence, and understanding.

In the matched cohort design, we compared the intervention and matched comparison groups’ baseline characteristics using two-sample *t* tests (or nonparametric alternatives) for numerical variables and chi-square tests for categorical variables. We used chi-square tests to compare rates of incident dialysis overall, by modality, and by setting across study groups for the nine-month follow-up. We also used two-sample *t* tests to compare the most recent eGFRs before dialysis start, a Wilcoxon rank sum test to compare days to dialysis start, and chi-square tests to compare statin and RAS inhibitor use at six to nine months after baseline. We used a conditional logistic regression model to explore the odds of starting outpatient dialysis within nine months of baseline across study groups.

In the post hoc analysis with full follow-up, we used a cause-specific Cox proportional hazards model to estimate differences in dialysis starts and outpatient dialysis starts between study groups. Individuals were censored when the follow-up time period ended or they switched to the Cricket intervention, died, had a kidney transplant, or started dialysis.

## Results

### Participant Characteristics

**Figure 1:**
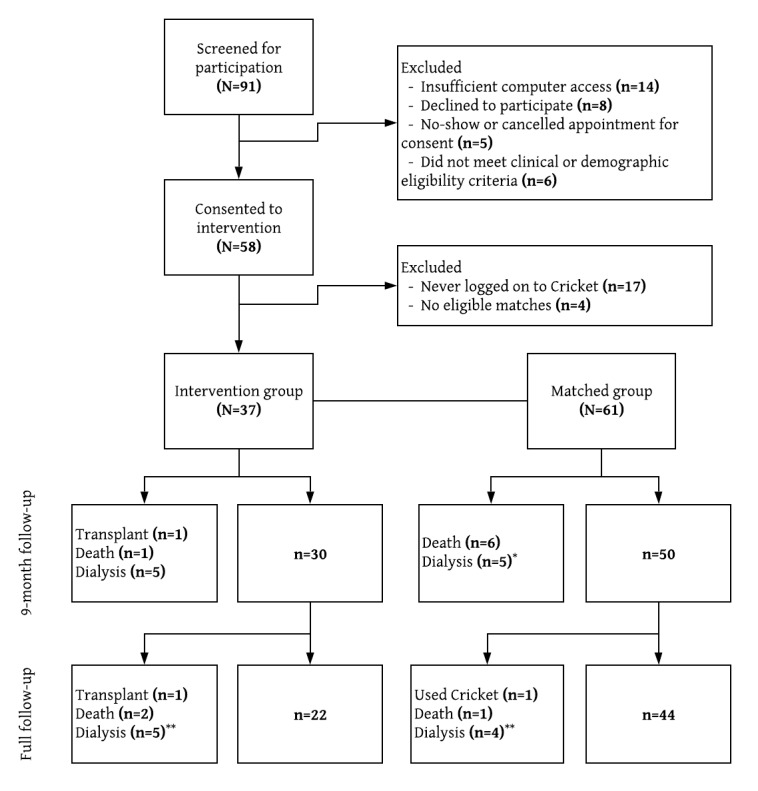
Patient flow diagram. *Two patients later died. **One patient later died.

Of the 91 patients screened, 58 patients met the eligibility criteria and consented to the intervention (Figure 1). Among these, we were unable to identify eligible matched comparisons for four, and another 17 patients never logged in to the Cricket platform, resulting in a total sample size of 37 participants in the intervention group. There were no significant characteristic differences between the 17 who never logged in to Cricket and those who did (Multimedia Appendix 2). A total of 61 patients were identified for the matched comparison group; 24 intervention participants had two matched comparators (as intended) and 13 had only one matched comparator. The intervention and comparison groups were largely similar in demographic and clinical characteristics at study baseline (Table 1).

**Table 1:** Baseline characteristics of participants.

Characteristic		Intervention group (n=37)	Comparison group (n=61)	*P* value^a^
Age (years), mean (SD)		67.2 (10.4)	68.8 (9.5)	.43
Gender (female), n (%)		25 (68)	41 (67)	>.99
**Race/ethnicity, n (%)**				**>.99**
	White non-Hispanic/Latino	35 (95)	59 (97)	
	Asian non-Hispanic/Latino	1 (3)	1 (2)	
	Unknown	1 (3)	1 (2)	
**Insurance type, n (%)**				**.55**
	Medicaid	3 (8)	9 (15)	
	Medicare	26 (70)	37 (61)	
	Commercial	8 (22)	15 (25)	
Diabetes, n (%)		20 (54)	35 (57)	.91
Hemoglobin A_1c_ <7%,^b^ n (%)		7 (35)	19 (54)	.27
Congestive heart failure, n (%)		8 (22)	15 (25)	.93
Chronic obstructive pulmonary disease, n (%)		5 (14)	9 (15)	>.99
Coronary artery disease, n (%)		8 (22)	11 (18)	.86
Blood pressure control <140/<90,^c^ n (%)		26 (72)	31 (67)	.81
Statin prescribed within 3 months of baseline, n (%)		27 (73)	34 (56)	.14
Renin-angiotensin system inhibitors prescribed within 3 months of baseline (%)		20 (54)	22 (36)	.13
Baseline albumin,^d^ mean (SD)		4.0 (0.4)	3.9 (0.5)	.44
Baseline eGFR,^e^ mean (SD)		19 (6)	21 (5)	.17

^a^From two-sample *t* tests or nonparametric alternatives for numerical variables and from chi-square tests for categorical variables, comparing intervention with comparison groups.

^b^For diabetic patients with A_1c_ values recorded within 90 days of baseline (intervention group: n=20; comparison group: n=35).

^c^For patients with blood pressure measured within 90 days of baseline (intervention group: n=36; comparison group: n=46).

^d^For patients with blood albumin measured within 90 days of baseline (intervention group: n=36; comparison group: n=56).

^e^For patients with glomerular filtration rate (GFR) measured within 90 days of baseline (intervention group: n=36; comparison group: n=44).

### Survey Results for Intervention Participants

Twenty-two of 37 intervention participants (59%) completed both a preprogram and posteducation survey. The educational phase of the online program was associated with significantly increased knowledge of CKD and increased interest in home treatment modalities (Table 2). Specifically, before education, 45% (10/22) of participants were unable to choose a dialysis modality. After education, 91% (20/22) of respondents made a choice, of whom 68% (15/22) preferred a home modality.

The intervention was very well-liked by the patients. Seventeen of 22 participants (77%) agreed or strongly agreed that the dialysis options education program was valuable in helping them make a treatment choice. When asked to rate their likeliness to recommend the Cricket Health program to a friend or family member (0 being not at all likely and 10 being extremely likely), the average response was 8.8 with 18 of 22 participants (82%) rating it 8 or higher. The survey asked participants to choose three features of the program that they found most valuable. Results showed that the most valued resources in order were the one-on-one nurse discussions (73%, 16/22), the educational videos (73%, 16/22), the frequently asked questions (55%, 12/22), discussion with mentors (41%, 9/22), and discussion with patient peers (36%, 8/22). The treatment preferences report and group exercises were not highly valued (14%, 3/22 and 5%, 1/22, respectively).

**Table 2:** Intervention group knowledge, confidence, and modality choice before and after the care program (n=22).^a^

Survey item		Preeducation	Posteducation	*P* value^b^
Percent of questions correct on survey of 7 knowledge-based questions (%), mean (SD)		52 (29)	94 (14)	<.001
**First intended type of dialysis, n (%)**				**.047**
	Home hemodialysis	2 (9)	3 (14)	
	Peritoneal dialysis	6 (27)	12 (55)	
	In-center hemodialysis	4 (18)	5 (23)	
	I don’t know	10 (45)	2 (9)	
**Agreed or strongly agreed with the following statement, n (%)**				
	I am afraid that my treatment would not be as good if I was responsible for my dialysis	5 (23)	3 (14)	.72
	I am confident that I could learn how to do self-care dialysis	15 (68)	19 (86)	.22
	I don’t understand self-care dialysis	9 (41)	3 (14)	.08
	I don’t see the point of doing dialysis myself when I can have a nurse do it	5 (23)	6 (27)	>.99

^a^Limited to n=22 intervention group patients with both pre- and posteducation results.

^b^From Wilcoxon signed rank test for average percent correct, exact symmetry tests for intended type of dialysis, and McNemar chi-square tests for all others.

### Clinical Outcomes

During the nine months of follow-up, one participant in the intervention group received a preemptive transplant, and six participants in the intervention group and one in the control group died before any dialysis start. Of the remaining participants, five in each group started dialysis; this difference was not statistically significant (between-group *P*=.49) (Table 3). Two of the dialysis patients in the control group later died before the end of the nine months of follow-up.

**Table 3:** Outcomes at nine months after baseline.^a^

Outcome		Intervention group (n=37)	Comparison group (n=61)	*P* value^b^
Deceased, n (%)		1 (3)	8 (13)	.15
Kidney transplant, n (%)		1 (3)	0 (0)	.80
Started dialysis, n (%)		5 (14)	5 (8)	.62
**Location of dialysis start, n (%)**				**.21**
	Outpatient	4 (80)	1 (20)	
	Inpatient	1 (20)	4 (80)	
	Dialysis type, n (%)			>.99
	In-center hemodialysis	3 (60)	4 (80)	
	Peritoneal dialysis	2 (40)	1 (20)	
	Home hemodialysis	0 (0)	0 (0)	
Last-recorded eGFR^c^ before dialysis start, mean (SD)		9.2 (4.0)	8.4 (2.1)	.70
Days from baseline to dialysis start, median (range)		183 (64-256)	154 (14-258)	>.99
RAS^d^ inhibitors prescribed 6-9 months from baseline, n (%)		20 (54)	16 (26)	.01
Statin prescribed 6-9 months from baseline, n (%)		26 (70)	36 (59)	.28
Blood pressure control <140/<90, n (%)		19 (51)	30 (49)	>.99

^a^Patients may be counted multiple times (eg, a patient who started dialysis and then died).

^b^From two-sample *t* tests for eGFR, Wilcoxon rank sum test for days to dialysis start, and chi-square tests for all other variables.

^c^eGFR: estimated glomerular filtration rate.

^d^RAS: renin-angiotensin system.

The intervention group had more frequent planned outpatient dialysis starts (80%, 4/5 versus 20%, 1/5) and dialysis starts using a home modality (40%, 2/5 versus 20%, 1/5) compared with the control group. There were no differences in the most recent eGFR before dialysis, median days from baseline to dialysis start, or blood pressure control (Table 3). In a conditional logistic regression model, intervention patients were 6.28 times more likely to start dialysis outpatient (planned) compared with control patients, although this difference did not reach statistical significance (OR 6.28, 95% CI 0.69-57.22).

In the post hoc analysis with full follow-up, the median follow-up time was 471 days (15.7 months), with a minimum of 351 days (11.7 months) and a maximum of 542 days (18.1 months). During this extended timeframe, one additional intervention participant received a preemptive transplant, and two participants in the intervention group and one in the control group died before any dialysis start. Nine additional participants started dialysis for a total of 19 participants starting dialysis within the full follow-up: 10 (27%) of 37 participants in the intervention group and 9 (15%) of 62 participants in the comparison group (Table 4). Among these patients, those in the intervention group were more likely to start on peritoneal dialysis than those in the comparison group (40%, 4/10 versus 11%, 1/9). Intervention participants were also more likely to start dialysis as a planned outpatient compared with the comparison group (80%, 8/10 versus 22%, 2/9). Two dialysis participants, one in each group, died before the end of full follow-up. A cause-specific Cox proportional hazards model showed no difference in dialysis starts between intervention and comparison participants (hazard ratio [HR] 1.89, 95% CI 0.76-4.65). However, intervention participants were significantly more likely to start dialysis in an outpatient setting compared with control (HR 6.89, 95% CI 1.46-32.66).

**Table 4:** Outcomes using all follow-up data.^a^

Outcome		Intervention group (n=37), n (%)	Comparison group (n=61), n (%)
Deceased		4 (11)	10 (16)
Kidney transplant		2 (5)	0 (0)
Started dialysis		10 (27)	9 (15)
**Location of dialysis start**			
	Outpatient	8 (80)	2 (22)
	Inpatient	2 (20)	7 (78)
**Dialysis type**			
	In-center hemodialysis	6 (60)	8 (89)
	Peritoneal dialysis	4 (40)	1 (11)
	Home hemodialysis	0 (0)	0 (0)

^a^Patients may be counted multiple times (eg, a patient who started dialysis and then died).

## Discussion

We found that a digital, virtual program of multidisciplinary care to support the management of patients with advanced CKD is feasible to implement with high levels of patient satisfaction. Moreover, we found that the program can improve patient knowledge about CKD and increase interest in home dialysis modalities. The program holds promise to increase outpatient dialysis starts as we found a higher likelihood of starting dialysis as an outpatient in the intervention group compared with controls in extended post hoc follow-up. A larger study with a longer follow-up time is needed to understand the degree to which the program improves clinical outcomes and reduces costs.

Our findings have important implications for the care of persons with kidney disease. Up to 35% of persons transitioning to dialysis have had no or little ongoing nephrology care, and more than half require hospitalization to initiate dialysis [1]. “Crashing” into dialysis is associated with high costs and higher rates of adverse clinical outcomes and hospitalizations after dialysis start [1]. Achieving a more orderly transition to dialysis with time for education and outpatient starts as well the use of home therapies has the potential to reduce costs, improve quality of life, and improve health outcomes [1,19]. Consistent with prior work [8], our study suggests there is a higher interest in home modalities after education on peritoneal and home hemodialysis. Our findings are also in accordance with randomized trials showing that multidisciplinary care for persons with advanced can improve outcomes. We extend the findings from those studies, which required increased staff and had limited scalability, to show that an online program is feasible to implement. We also validate our prior findings [10] and show the value of this educational program in a rural, community-based setting. Our study adds to the importance of multimodal education, including videos, written content, and chats, because these resources were found useful by program participants.

There are several lessons learned that require consideration. Having to use physician-led adjudication to ensure the quality of dialysis outcome ascertainment has important implications for future research studies. The gold standard for incident ESRD assessment has been linkage to the United States Renal Data System (USRDS), but that is not practical when evaluating these interventions in real life and with short follow-up times. We found using only codes was insufficient to characterize disease, as has been previously reported [20]. Although new data are becoming available on building EHR-based kidney disease phenotypes [14], future studies need to incorporate quality control and validation of measures and outcomes around dialysis starts. We also learned about the limitations of virtual programs to reach all patients, particularly those with limited internet access. Because of these findings, Cricket Health added a telephonic program. Our findings show that it is imperative to evaluate these interventions in real-world settings.

These results are subject to additional limitations. This was an observational study, so unmeasured confounders may remain. As such, it is possible that unobserved differences between our intervention group and the comparison group influenced the results. We mitigated this potential bias to the best of our ability by matching demographic and clinical criteria, although we were unable to match as closely or cluster within a provider because of the relatively small size of our study site. The study was designed to understand dialysis modality choice and did not systematically assess transplant interest or conservative care choice. The study population was mostly white, and future studies should be deployed in populations with wider race and ethnic representation. However, a majority of patients were covered by Medicare or Medicaid insurance, and the patient characteristics are similar to national data. Additionally, because this was designed as a pilot study, a power analysis was not conducted before data collection and analysis. Therefore, our analyses may not be adequately powered to detect meaningful differences.

In conclusion, a virtual multidisciplinary care program for persons with advanced CKD was shown to improve patient CKD knowledge, confidence in self-care, and interest in home dialysis therapies. Our findings also suggest this virtual multidisciplinary care program may increase the likelihood of starting dialysis in a planned manner in the outpatient setting. Larger studies are required to evaluate the impact of virtual programs in improving outcomes and reducing costs.

## Appendix

Multimedia Appendix 1 Cricket Health surveys.

Multimedia Appendix 2 Demographic characteristics by enrollment status among patients who consented to the study.

## Abbreviations

CKD: chronic kidney disease
eGFR: estimated glomerular filtration rate
EHR: electronic health record
ESRD: end-stage renal disease
GFR: glomerular filtration rate
RAS: renin-angiotensin system
