# Supplementary material for: A Virtual Multidisciplinary Care Program for Management of Advanced Chronic Kidney Disease: Matched Cohort Study
Source: J Med Internet Res. 2020 Feb 12;22(2):e17194. doi: 10.2196/17194 (PMC7055849; doi:10.2196/17194)
Supplement: Multimedia Appendix 1 [file jmir_v22i2e17194_app1.docx]

| Cricket Health Pre-study Survey |  |
| --- | --- |
|  |  |
| **Patient ID:**  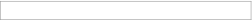  1. How have you learned about chronic kidney disease?  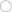 Personal research (online, library)  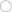 Kidney disease education program  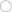 Conversations with my doctor or healthcare provider 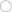 Other (please specify)  2. When were you diagnosed with chronic kidney disease?  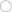 <1 yr ago  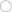 1-2 years ago 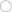 2-5 years ago 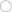 More than 5 years ago  3. Which of the following is a form of in-home treatment that uses patient’s abdomen (belly area) to remove toxins from the body?  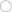 Conservative management  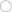 Kidney transplant  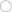 Peritoneal dialysis (PD)  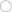 Home Hemodialysis (HHD)  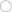 In-Center Hemodialysis (IHD)  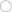 I don’t know  4. Which of the following is a form of in-home treatment that uses a machine to filter blood outside of the body?  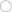 Conservative management  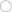 Kidney transplant  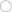 Peritoneal dialysis (PD)  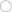 Home Hemodialysis (HHD)  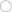 In-Center Hemodialysis (IHD)  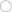 I don’t know  5. Which of the following is a form of treatment where healthcare professionals use a machine to filter blood in the clinic?  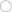 Conservative management  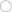 Kidney transplant  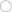 Peritoneal dialysis (PD)  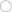 Home Hemodialysis (HHD)  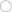 In-Center Hemodialysis (IHD)  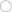 I don’t know  6. Which of the following is a form of medical treatment to keep the patient as comfortable as possible for as long as possible, without dialysis?  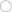 Conservative management  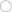 Kidney transplant  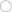 Peritoneal dialysis (PD)  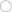 Home Hemodialysis (HHD)  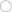 In-Center Hemodialysis (IHD)  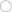 I don’t know  7. Which of the following is a form of treatment in which a patient undergoes a surgery to receive a donated kidney?  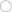 Conservative management  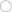 Kidney transplant  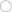 Peritoneal dialysis (PD)  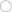 Home Hemodialysis (HHD)  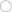 In-Center Hemodialysis (IHD)  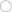 I don’t know  8. Which type of dialysis does NOT require needles?  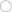 Peritoneal dialysis (PD)  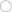 Home Hemodialysis (HHD)  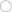 In-Center Hemodialysis (IHD)  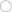 I don’t know  9. Which dialysis treatment options allow patients to travel?  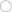 Peritoneal dialysis (PD)  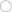 Home Hemodialysis (HHD)  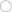 In-Center Hemodialysis (IHD)  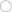 All of the above  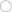 None of the above, dialysis patients cannot travel  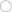 I don’t know  10. Please indicate your level of agreement with the following statements:  I AM AFRAID that my treatment would not be as good if I was responsible for my dialysis.  Strongly disagree, Disagree, Neutral or unsure, Agree, Strongly agree  I AM CONFIDENT that I could learn how to do self-care dialysis.  Strongly disagree, Disagree, Neutral or unsure, Agree, Strongly agree  I DON’T understand self-care dialysis.  Strongly disagree, Disagree, Neutral or unsure, Agree, Strongly agree  I DON’T SEE THE POINT of doing dialysis myself when I can have a nurse do it.  Strongly disagree, Disagree, Neutral or unsure, Agree, Strongly agree  I feel ready to choose a treatment option that would be best for me if I experience kidney failure.  Strongly disagree, Disagree, Neutral or unsure, Agree, Strongly agree  The Options Education program was valuable to me in making my treatment choice.  Strongly disagree, Disagree, Neutral or unsure, Agree, Strongly agree  11. If I need dialysis, my intended first type of dialysis is: Peritoneal dialysis (PD)  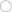 Peritoneal dialysis (PD)  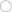 Home Hemodialysis (HHD)  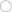 In-Center Hemodialysis (IHD)  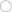 I don’t know  12. Please indicate what type of dialysis you would NOT consider at present (check all that apply):  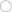 Peritoneal dialysis (PD)  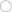 Home Hemodialysis (HHD)  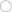 In-Center Hemodialysis (IHD)  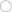 I don’t know |  |

| Cricket Health Post-education Survey |  |
| --- | --- |
|  |  |
| * 1. **Your ID:**  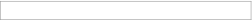  2. Which of the following is a form of in-home treatment that uses patient’s abdomen (belly area) to remove toxins from the body?  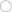 Conservative management  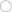 Kidney transplant  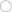 Peritoneal dialysis (PD)  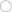 Home Hemodialysis (HHD)  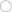 In-Center Hemodialysis (IHD)  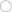 I don’t know  3. Which of the following is a form of in-home treatment that uses a machine to filter blood outside of the body?  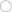 Conservative management  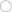 Kidney transplant  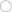 Peritoneal dialysis (PD)  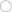 Home Hemodialysis (HHD)  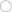 In-Center Hemodialysis (IHD)  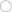 I don’t know  4. Which of the following is a form of treatment where healthcare professionals use a machine to filter blood in the clinic?  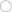 Conservative management  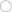 Kidney transplant  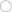 Peritoneal dialysis (PD)  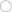 Home Hemodialysis (HHD)  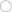 In-Center Hemodialysis (IHD)  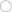 I don’t know  5. Which of the following is a form of medical treatment to keep the patient as comfortable as possible for as long as possible, without dialysis?  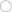 Conservative management  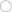 Kidney transplant  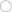 Peritoneal dialysis (PD)  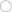 Home Hemodialysis (HHD)  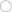 In-Center Hemodialysis (IHD)  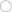 I don’t know  6. Which of the following is a form of treatment in which a patient undergoes a surgery to receive a donated kidney?  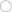 Conservative management  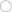 Kidney transplant  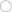 Peritoneal dialysis (PD)  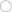 Home Hemodialysis (HHD)  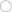 In-Center Hemodialysis (IHD)  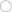 I don’t know  7. Which type of dialysis does NOT require needles?  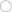 Peritoneal dialysis (PD)  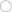 Home Hemodialysis (HHD)  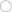 In-Center Hemodialysis (IHD)  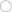 I don’t know  8. Which dialysis treatment options allow patients to travel?  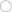 Peritoneal dialysis (PD)  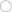 Home Hemodialysis (HHD)  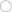 In-Center Hemodialysis (IHD)  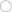 All of the above  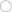 None of the above, dialysis patients cannot travel  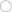 I don’t know  9. Please indicate your level of agreement with the following statements:  I AM AFRAID that my treatment would not be as good if I was responsible for my dialysis.  Strongly disagree, Disagree, Neutral or unsure, Agree, Strongly agree  I AM CONFIDENT that I could learn how to do self-care dialysis.  Strongly disagree, Disagree, Neutral or unsure, Agree, Strongly agree  I DON’T understand self-care dialysis.  Strongly disagree, Disagree, Neutral or unsure, Agree, Strongly agree  I DON’T SEE THE POINT of doing dialysis myself when I can have a nurse do it.  Strongly disagree, Disagree, Neutral or unsure, Agree, Strongly agree  I feel ready to choose a treatment option that would be best for me if I experience kidney failure.  Strongly disagree, Disagree, Neutral or unsure, Agree, Strongly agree  The Options Education program was valuable to me in making my treatment choice.  Strongly disagree, Disagree, Neutral or unsure, Agree, Strongly agree  10. If I need dialysis, my intended first type of dialysis is: Peritoneal dialysis (PD)  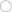 Peritoneal dialysis (PD)  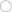 Home Hemodialysis (HHD)  In-Center Hemodialysis (IHD)  I don’t know  11. Please indicate what type of dialysis you would NOT consider at present (check all that apply):  Peritoneal dialysis (PD)  Home Hemodialysis (HHD)  In-Center Hemodialysis (IHD)  I don’t know  12. Choose **three** features of Cricket Health that you found most valuable:  Educational Videos  Frequently Asked Questions  Discussion with Patient Peers  Group Exercises  1-on-1 Nurse Discussion  Discussion with Mentors  Treatment Preferences Report  13. Based on your experience, how likely are you to recommend Cricket Health program to a friend or family member should they need it? (0 being not at all likely and 10 being extremely likely) |  |
