# Supplementary material for: A Virtual Multidisciplinary Care Program for Management of Advanced Chronic Kidney Disease: Matched Cohort Study
Source: J Med Internet Res. 2020 Feb 12;22(2):e17194. doi: 10.2196/17194 (PMC7055849; doi:10.2196/17194)
Supplement: Multimedia Appendix 2 [file jmir_v22i2e17194_app2.docx]

Table 1. Demographic characteristics by enrollment status among patients who consented to the study.

| Characteristic | Consented but never enrolled  (N=17) | Consented and enrolled (intervention group) (N=37) | *P*-value^e^ |
| --- | --- | --- | --- |
| Age, mean (SD) | 67 (13.6) | 67 (10.4) | .95 |
| Gender (female), n (%) | 11 (65) | 25 (68) | >.99 |
| Race/ethnicity, n (%) |  |  | .58 |
| White non-Hispanic/Latino | 15 (88) | 35 (95) |  |
| Asian non-Hispanic/Latino | 1 (6) | 1 (3) |  |
| Other non-Hispanic/Latino | 1 (6) | 0 (0) |  |
| Other Hispanic/Latino | 0 (0) | 0 (0) |  |
| Unknown | 0 (0) | 1 (3) |  |
| Insurance type, n (%) |  |  | .88 |
| Medicaid | 1 (6) | 3 (8) |  |
| Medicare | 11 (65) | 26 (70) |  |
| Commercial | 5 (29) | 8 (22) |  |
| Diabetes, n (%) | 7 (41) | 20 (54) | .57 |
| Hemoglobin A1c <7%^a^, n (%) | 1 (22) | 7 (35) | .38 |
| Congestive heart failure, n (%) | 4 (24) | 8 (22) | >.99 |
| Chronic obstructive pulmonary disease, n (%) | 4 (24) | 5 (14) | .44 |
| Coronary artery disease, n (%) | 3 (18) | 8 (22) | >.99 |
| Median number of nephrology visits in past 12 months (IQR) | 3 (4-3) | 3 (4-3) | .45 |
| Blood pressure control <140/<90^b^, n (%) | 10 (59) | 26 (70) | .36 |
| Baseline Albumin,^c^  mean (SD) | 4.1 (0.4) | 4.0 (0.4) | .16 |
| Baseline estimated glomular filtration rate,^d^ mean (SD) | 21.4 (7.7) | 19.1 (5.6) | .26 |

^a^ For diabetic participants with A1c values recorded within 90 days of baseline (intervention group n=20, non-enrolled group n=7)

^b^ For participants with blood pressure measured within 90 days of baseline (intervention group n=36, non-enrolled group n=17)

^c^ For participants with blood albumin measured within 90 days of baseline (intervention group n=36, non-enrolled group n=17)

^d^ For participants with estimate glomular filtration rate measured within 90 days of baseline (intervention group n=36, non-enrolled group n=17)

^e^ From two sample t-tests or non-parametric alternatives for numerical variables and from chi-squared tests for categorical variables, comparing intervention to comparison group
